# Examination of Changes in Health Status Among Michigan Medicaid Expansion Enrollees From 2016 to 2017

**DOI:** 10.1001/jamanetworkopen.2020.8776

**Published:** 2020-07-10

**Authors:** Minal R. Patel, Renuka Tipirneni, Edith C. Kieffer, Jeffrey T. Kullgren, John Z. Ayanian, Tammy Chang, Erica Solway, Erin Beathard, Matthias Kirch, Sunghee Lee, Sarah Clark, Jennifer Skillicorn, Zachary Rowe, Susan D. Goold

**Affiliations:** 1 Department of Health Behavior and Health Education, School of Public Health, University of Michigan, Ann Arbor; 2 Institute for Healthcare Policy and Innovation, University of Michigan, Ann Arbor; 3 Department of Internal Medicine, University of Michigan, Ann Arbor; 4 School of Social Work, University of Michigan, Ann Arbor; 5 US Department of Veterans Affairs VA Ann Arbor Healthcare System, Ann Arbor, Michigan; 6 Center for Bioethics and Social Sciences in Medicine, University of Michigan, Ann Arbor; 7 Gerald R. Ford School of Public Policy, University of Michigan, Ann Arbor; 8 Department of Health Management and Policy, School of Public Health, University of Michigan, Ann Arbor; 9 Department of Family Medicine, University of Michigan, Ann Arbor; 10 Institute for Social Research, University of Michigan, Ann Arbor; 11 Department of Pediatrics, University of Michigan, Ann Arbor; 12 Friends of Parkside, Detroit, Michigan

## Abstract

**Importance:**

Evidence about the health benefits of Medicaid expansion has been mixed and has largely come from comparing expansion and nonexpansion states.

**Objective:**

To examine the self-reported health of enrollees in Michigan’s Medicaid expansion, the Healthy Michigan Plan (HMP), over time.

**Design, Setting, and Participants:**

A telephone survey from January 1 to October 31, 2016 (response rate, 53.7%), and a follow-up survey from March 1, 2017, to January 31, 2018 (response rate, 83.4%), were conducted in Michigan, which expanded Medicaid in 2014 through a Section 1115 waiver permitting state-specific modifications. Four thousand ninety HMP beneficiaries aged 19 to 64 years with at least 12 months of HMP coverage and at least 9 months in a Medicaid health plan were eligible to participate. Data were analyzed from April 1 to November 30, 2018.

**Main Outcomes and Measures:**

Surveys measured demographic characteristics and health status. Analyses included weights for sampling probability and nonresponse. Comparisons between 2016 and 2017 included those who responded to both surveys (n = 3097).

**Results:**

Of the 3097 respondents to the 2017 follow-up survey, 2388 (77.1%) were still enrolled in HMP (current enrollees) and 709 (22.9%) were no longer enrolled when surveyed (former enrollees). Among all follow-up respondents, a weighted 37.5% (95% CI, 35.3%-39.9%) were aged 19 to 34 years, 34.0% (95% CI, 31.8%-36.2%) were aged 35 to 50 years, and 28.5% (95% CI, 26.7%-30.3%) were aged 51 to 64 years; 53.0% (95% CI, 50.8%-55.3%) were female. Respondents who reported fair or poor health decreased from 30.7% (95% CI, 28.7%-32.8%) in 2016 to 27.0% (95% CI, 25.1%-29.0%) in 2017 (adjusted odds ratio [AOR], 0.66 [95% CI, 0.53-0.81]; *P* < .001), with the largest decreases observed in respondents who were non-Hispanic black (from 31.5% [95% CI, 27.1%-35.9%] in 2016 to 26.0% [95% CI, 21.9%-30.1%] in 2017; *P* = .009), from the Detroit metropolitan area (from 30.7% [95% CI, 27.0%-34.4%] in 2016 to 24.9% [95% CI, 21.6%-28.3%] in 2017; *P* = .001), and with an income of 0% to 35% of the federal poverty level (from 37.6% [95% CI, 34.2%-40.9%] in 2016 to 32.3% [95% CI, 29.1%-35.5%] in 2017; *P* < .001). From 2016 to 2017, the mean number of days of poor physical health in the past month decreased significantly from 6.9 (95% CI, 6.5-7.4) to 5.7 (95% CI, 5.3-6.0) (coefficient, −6.10; *P* < .001), including among current (from 7.0 [95% CI, 6.5-7.5] to 5.6 [95% CI, 5.1-6.0]; *P* < .001) and former (from 6.8 [95% CI, 5.9-7.7] to 5.8 [95% CI, 5.0-6.7]; *P* = .02) enrollees, those with 2 or more chronic conditions (from 9.9 [95% CI, 9.3-10.6] to 8.5 [95% CI, 7.8-9.1]; *P* < .001), across all age groups (19-34 years, from 4.3 [95% CI, 3.7-4.9] to 3.0 [95% CI, 2.5-3.5]; *P* < .001; 35-50 years, from 8.2 [95% CI, 7.3-9.0] to 6.9 [95% CI, 6.1-7.7]; *P* = .002; 51-64 years, from 9.0 [95% CI, 8.2-9.8] to 7.6 [95% CI, 6.9-8.3]; *P* = .001), and among non-Hispanic white (from 7.5 [95% CI, 7.0-8.1] to 6.1 [95% CI, 5.6-6.6]; *P* < .001) and black (from 5.9 [95% CI, 5.1-6.8] to 4.4 [95% CI, 3.6-5.1]; *P* < .001) respondents. No changes in days of poor mental health or usual activities missed owing to poor physical or mental health were observed.

**Conclusions and Relevance:**

These findings suggest that HMP enrollees in Michigan have experienced improvements in self-reported health over time, including minority groups with a history of health disparities and enrollees with chronic health conditions.

## Key Points

**Question:**

What longitudinal changes in self-reported health status and days of poor health among racial, ethnic, urban/rural, and very-low-income subgroups of enrollees are associated with Medicaid expansion?

**Findings:**

In this survey study of 3097 respondents, reports of fair or poor health and days of poor physical health decreased over time among enrollees, especially among non-Hispanic black enrollees and those with very low incomes. There were no statistically significant differences in the number of days of poor mental health or the number of days of usual activities missed owing to poor physical or mental health over time.

**Meaning:**

These findings suggest that within Medicaid expansion, the health of vulnerable populations is improving.

## Teaser

This survey study examines the self-reported health of enrollees in Michigan’s Medicaid expansion, the Healthy Michigan Plan, among respondents to surveys in 2016 and 2017.

## Introduction

Under the Patient Protection and Affordable Care Act (ACA), Medicaid eligibility expanded to provide coverage to a greater portion of the low-income, nonelderly adult population. To date, 36 states and the District of Columbia have expanded their Medicaid programs.^1^ Several studies have shown that expanding Medicaid has improved access to primary care and medical homes,^2^ increased uptake of preventive services,^3,4^ and reduced disparities in insurance coverage across multiple demographic groups, including by race, marital status, and age.^5,6^ Racial/ethnic minority groups have experienced the largest gains in coverage as a result of the ACA, especially the Latino/Hispanic population.^7^

Research examining the association between having insurance coverage and improved health has been mixed but more recently suggests an association with health improvement.^8,9,10^ In studies examining expansions in health insurance under the ACA, reductions in emergency department use and improved self-reported health were observed among enrollees 1 year after expansion,^11,12^ and improved survival has also been noted among individuals with end-stage kidney disease.^13^

Medicaid expansion in particular may have large health effects, because the Medicaid expansion specifically targets low-income populations that have been historically underinsured or uninsured, have been medically underserved, and have more chronic health conditions.^14^ Most state and national studies examining the effect of Medicaid expansion on health have compared health outcomes for low-income persons in expansion and nonexpansion states. These studies have found mixed results, with some findings suggesting reductions in poor health days in expansion states^15,16,17^ and other findings suggesting no changes in self-reported health status.^3,4^

To our knowledge, no longitudinal studies of the health status of actual Medicaid expansion enrollees to learn whether those who enrolled experience improved health over time have been published to date. Health in the United States is patterned strongly along both socioeconomic and racial/ethnic lines,^18^ and Medicaid plays a large role in providing health care coverage to low-income racial/ethnic minority populations that would otherwise be uninsured.^7^ Medicaid expansion reaches a broader population of low-income individuals, such as childless adults and those with incomes of 100% to 138% of the federal poverty level (FPL), who have been historically underserved. Given national goals to eliminate health disparities within racial/ethnic minority groups^19^ and continuing debate over Medicaid expansion in general, it is important to understand the effect of Medicaid expansion on changes in health status among populations that have historically been underserved.

The enrollee surveys conducted as part of the evaluation of Michigan’s Medicaid expansion program provide a valuable opportunity to examine changes in health over time within high-priority demographic subgroups as a result of Medicaid expansion. Michigan received a Section 1115 waiver from the Centers for Medicare & Medicaid Services that allowed the implementation of an alternative model to Medicaid expansion under the ACA.^20^ Michigan’s approach, the Healthy Michigan Plan (HMP), began enrolling beneficiaries at 133% or less of the FPL in April 2014. As of March 2019, more than 675 000 low-income adults (aged 19-64 years) were enrolled. The HMP covers essential health benefits required by the ACA and benefits such as dental and vision care, home health services, and family planning services. Michigan has documented disparities in health by race/ethnicity, income, and geography.^21^ The purpose of this study was to examine longitudinal changes in enrollees’ self-reported health status and days of poor health over time in racial, ethnic, urban/rural, and very-low-income subgroups.

## Methods

### Study Design, Data Sources, and Study Population

This study used 2 waves of survey data gathered after HMP implementation in April 2014. The surveys were administered as part of an evaluation of HMP for the Michigan Department of Health and Human Services and the Centers for Medicare & Medicaid Services. As an evaluation of a public program, the University of Michigan and Michigan Department of Health and Human Services institutional review boards deemed the study exempt; therefore, no informed consent was required. We told all of those who were selected to participate that their participation was voluntary and asked for their verbal permission to continue with the survey. This study followed the American Association for Public Opinion Research (AAPOR) reporting guideline.

An initial telephone survey was administered to HMP enrollees between January 1 and October 31, 2016, 2 years after HMP implementation. The sample included enrollees aged 19 to 64 years with HMP enrollment at least 12 months before sampling and at least 9 months in an HMP managed care plan; preferred language of English, Spanish, or Arabic; and a complete Michigan address and telephone number in the state’s enrollment files. We used random sampling stratified by income and geographic region and conducted telephone interviews with enrollees. Additional survey methods have been described elsewhere.^22,23^ A total of 9227 eligible HMP enrollees were mailed a letter and brochure describing the project before receiving a telephone call. The 2016 study sample included 4108 HMP enrollees (weighted sample, 384 262 enrollees) who completed the survey. We excluded 18 surveys from the analysis owing to more than 20% missing data, leaving 4090 respondents with fully completed surveys (weighted response rate, 53.7% using the AAPOR response rate formula 3).^24^

Respondents in 2016 who consented to recontact (3957 [96.7%]) were eligible for the 2017 follow-up survey (March 1, 2017, to January 31, 2018). The response rate was 83.4%. The final sample for the analyses presented herein includes the 3097 HMP enrollees who responded to both surveys. Compared with nonrespondents, follow-up respondents were more likely to be older (aged 51-64 years, 1221 of 3194 [38.2% unweighted; 30.5% weighted as part of weight development process] vs 166 of 608 [27.3% unweighted; 20.3% weighted]), have lower income (0%-35% of FPL, 1223 of 3194 [38.3% unweighted; 53.1% weighted] vs 206 of 608 [33.9% unweighted; 45.6% weighted]), and have completed the initial survey in English (3063 of 3102 [98.7%] vs 569 of 608 [93.6%]) (eTable in the Supplement).

### Measures

The surveys measured demographic characteristics such as self-reported race/ethnicity and educational attainment, health status, access to and use of health care services, and health risks and behaviors using standard measures from established national surveys.^25,26,27^ Other demographic characteristics, including age, geographic region, and enrollment status, were obtained from the state’s Medicaid files. Enrollees with at least 1 chronic physical health condition were identified using Medicaid claims in the 24-month period before survey sampling, the 12-month period after survey sampling, and/or self-report in the 2016 or 2017 survey. Health status was assessed by the following survey items: “In general, would you say your health is…(excellent, very good, good, fair, poor [responses fair and poor were grouped together for this analysis])?” “For how many days during the past 30 days was your physical health not good?” “For how many days during the past 30 days was your mental health not good?” and “During the past 30 days, for how many days did poor physical or mental health keep you from doing your usual activities, such as self-care, work, or recreation?”

### Statistical Analysis

Data were analyzed from April 1 to November 30, 2018. Analysis included survey weights to adjust for probability of selection and nonresponse bias. Weighted proportions reflect results for the target population of all HMP enrollees. Descriptive statistics included weighted means and percentages. Mixed-effects logistic regression models were used to examine changes in fair or poor health across all respondents, subgroups, and select demographic characteristics (race/ethnicity, age, income, geographic region).^28^ Models adjustments were variables known to differentially affect health status over time and included chronic disease status and number, new diagnosis, age, sex, race/ethnicity, FPL category, educational level, and insurance status in the 12 months before HMP. Paired-sample *t* tests were used to assess changes in days of poor health between 2016 and 2017 among all respondents and subgroups of interest and by demographic characteristics. All analyses were conducted with STATA software, version 15 (StataCorp LLC), as exploratory and descriptive, with no formal adjustment for type I error. Two-sided *P* < .05 indicated significance.

## Results

### Study Population

Of 3097 respondents to the 2017 follow-up survey, 2388 (77.1% [95% CI, 74.9%-78.6%]) were still enrolled in HMP (current enrollees) and 709 (22.9% [95% CI, 21.4%-25.1%]) were no longer enrolled when surveyed (former enrollees). Among all follow-up respondents, 71.5% (95% CI, 69.7%-73.3%) were 50 years or younger; 53.0% (95% CI, 50.8%-55.3%) were female, and 47.0% (95% CI, 44.7%-49.2%) were male; 59.6% (95% CI, 57.4%-61.7%) reported white race; 19.9% (95% CI, 19.2%-20.7%) had income of greater than 100% of the FPL; 51.7% (95% CI, 49.4%-53.9%) had a high school education or less; 57.1% (95% CI, 55.0%-59.3%) were employed; 74.7% (95% CI, 72.6%-76.7%) reported having a chronic condition; and 42.9% (95% CI, 41.7%-44.1%) lived in the Detroit metropolitan area (Table 1).

**Table 1.** Demographic Characteristics of All Respondents

Characteristics	Unweighted No. (n = 3097)^a^	Weighted % (95% CI)
Age, y		
19-34	909	37.5 (35.3-39.9)
35-50	969	34.0 (31.8-36.2)
51-64	1219	28.5 (26.7-30.3)
Sex		
Male	1230	47.0 (44.7-49.2)
Female	1867	53.0 (50.8-55.3)
Race/ethnicity		
White, non-Hispanic	2058	59.6 (57.4-61.7)
Black, non-Hispanic	634	26.8 (24.8-28.9)
Hispanic	138	5.0 (4.1-6.0)
Other, non-Hispanic	228	8.7 (7.4-10.1)
FPL category, %		
0-35	1218	52.3 (51.2-53.5)
36-99	1084	27.7 (26.8-28.7)
≥100	795	19.9 (19.2-20.7)
Highest level of education attained		
Less than high school	355	11.2 (9.9-12.6)
High school graduate	1266	40.5 (38.3-42.8)
Some college	665	22.8 (20.9-24.9)
Associate’s degree	408	12.9 (11.5-14.5)
Bachelor’s degree	308	9.9 (8.6-11.3)
Postgraduate degree	88	2.7 (2.0-3.5)
Employed or self-employed	1742	57.1 (55.0-59.3)
Married	750	19.4 (17.9-20.9)
Chronic disease	2413	74.7 (72.6-76.7)
≥2 chronic conditions	1716	51.9 (49.7-54.2)
Region		
Northern Michigan	574	9.1 (8.7-9.5)
Central Michigan	980	29.6 (28.6-30.6)
Southern Michigan	633	18.4 (17.6-19.3)
Detroit metropolitan	910	42.9 (41.7-44.1)

Abbreviation: FPL, federal poverty level.

^a^ Owing to missing data, some subgroups may not total 3097.

### Changes in Self-reported Health by Demographic and Health Characteristics

**Figure 1.**
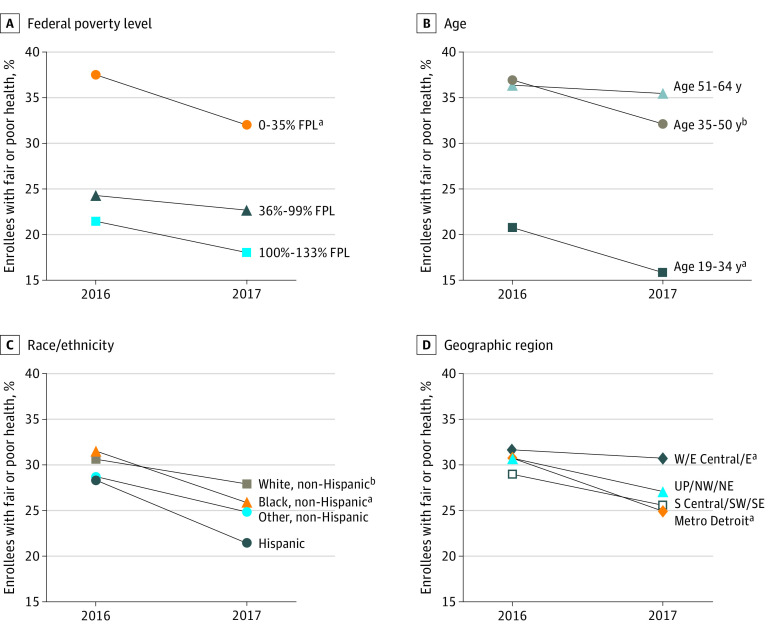
Changes Among All Enrollees in Fair or Poor Health From 2016 to 2017 by Select Demographic Characteristics Data are weighted for sampling probability and nonresponse. E indicates Eastern Michigan; E Central, Eastern Central Michigan; FPL, federal poverty level; Metro, metropolitan; NE, Northeastern Michigan; NW, Northwestern Michigan; S Central, Southern Central Michigan; SE, Southeastern Michigan; SW, Southwestern Michigan; UP, Upper Peninsula; and W, Western Michigan. Mixed-effects logistic regression models restricted to each group category were used to test for a significant change over time. ^a^*P* < .01. ^b^*P* < .05.

In 2017, 27.0% (95% CI, 25.1%-29.0%) of current and former enrollees reported fair or poor health, a change from the 30.7% (95% CI, 28.7%-32.8%) reporting fair or poor health in 2016 (adjusted odds ratio [AOR], 0.66 [95% CI, 0.53-0.81]) (Table 2). Significant reductions in reports of fair or poor health from 2016 to 2017 were observed among current and former enrollees who were non-Hispanic white (from 30.6% [95% CI, 28.1%-33.2%] to 27.9% [95% CI, 25.4%-30.3%]; *P* < .001), non-Hispanic black (from 31.5% [95% CI, 27.1%-35.9%] to 26.0% [95% CI, 21.9%-30.1%]; *P* < .001), from the Detroit metropolitan area (from 30.7% [95% CI, 27.0%-34.4%] to 24.9% [95% CI, 21.6%-28.3%]; *P* < .001), and with an income of 0% to 35% of the FPL (from 37.6% [95% CI, 34.2%-40.9%] to 32.3% [95% CI, 29.1%-35.5%]; *P* < .001) (Figure 1). Significant reductions in fair or poor health were also found for those aged 19 to 34 years (from 20.7% [95% CI, 17.4%-24.1%] to 15.9% [95% CI, 12.9%-18.8%]; *P* = .004) and aged 35 to 50 years (from 36.9% [95% CI, 33.1%-40.7%] to 32.1% [95% CI, 28.5%-35.8%]; *P* = .01) (Figure 1) and those with 2 or more chronic conditions (from 45.6% [95% CI, 42.7%-48.5%] to 40.9% [95% CI, 38.0%-43.8%]; *P* = .002) (Table 2).

**Table 2.** Changes in Fair or Poor Health Among All Respondents and Various Subgroups

Respondents	Fair or poor health				
	Survey year, weighted % (95% CI)		AOR (95% CI)	*P* value
	2016	2017		
All (n = 3097)^a^	30.7 (28.7-32.8)	27.0 (25.1-29.0)	0.66 (0.53-0.81)	<.001
Still enrolled (n = 2387)^b^	31.4 (29.1-33.8)	27.2 (25.0-29.4)	0.62 (0.48-0.79)	<.001
No longer enrolled and uninsured (n = 193)^b^	23.6 (16.9-31.9)	19.0 (12.8-27.3)	0.65 (0.29-1.41)	.27
≥2 chronic conditions (n = 1767)^b^	45.6 (42.7-48.5)	40.9 (38.0-43.8)	0.68 (0.53-0.86)	.002

Abbreviation: AOR, adjusted odds ratio.

^a^ Mixed-effects logistic regression model adjusted for presence of a chronic condition, age, sex, race/ethnicity, federal poverty level (FPL) category, educational level, and insurance status in 12 months before Healthy Michigan Plan.

^b^ Mixed-effects logistic regression model adjusted for new chronic condition diagnosis in 2017 (from 2016 to 2017), number of chronic conditions, age, sex, race/ethnicity, FPL category, and educational level.

### Changes in Days of Poor Health

From 2016 to 2017, the mean number of days enrollees reported poor physical health decreased (from 6.9 [95% CI, 6.5-7.4] to 5.7 [95% CI, 5.3-6.0]; coefficient, −6.10; *P* < .001). This decrease was seen in both current enrollees (from 7.0 [95% CI, 6.5-7.5] to 5.6 [95% CI, 5.1-6.0]; coefficient, −5.65; *P* < .001) and former enrollees (from 6.8 [95% CI, 5.9-7.7] to 5.8 [95% CI, 5.0-6.7]; coefficient, −2.30; *P* = .02) and those with 2 or more chronic conditions (from 9.9 [95% CI, 9.3-10.6] to 8.5 [95% CI, 7.8-9.1]; coefficient, −4.39; *P* < .001) (Table 3). No significant change was observed among those no longer enrolled and uninsured. There was also no statistically significant difference from 2016 to 2017 in the number of days of poor mental health or the number of days missed owing to poor physical or mental health (Table 3).

**Table 3.** Changes in Health Status Among Various Subgroups^a^

Respondents	Poor physical health			Poor mental health			Time missed owing to poor physical and mental health		
	Mean (95% CI), d	Difference (95% CI), d	*P* value^b^	Mean (95% CI), d	Difference (95% CI), d	*P* value	Mean (95% CI), d	Difference (95% CI), d	*P* value
All									
2016	6.9 (6.5 to 7.4)	–1.2 (–1.7 to –0.9)	<.001	6.0 (5.5 to 6.4)	–0.2 (–0.7 to 0.2)	.28	5.3 (4.8 to 5.7)	–0.3 (–0.6 to 0.2)	.28
2017	5.7 (5.3 to 6.0)			5.8 (5.4 to 6.3)			5.0 (4.7 to 5.4)		
Still enrolled									
2016	7.0 (6.5 to 7.5)	–1.4 (–1.9 to –0.9)	<.001	6.1 (5.5 to 6.6)	–0.3 (–0.9 to 0.1)	.12	5.2 (4.7 to 5.7)	0.2 (–0.7 to 0.2)	.31
2017	5.6 (5.1 to 6.0)			5.8 (5.3 to 6.3)			5.0 (4.6 to 5.4)		
No longer enrolled and uninsured									
2016	4.9 (3.4 to 6.3)	–1.1 (–2.5 to –0.6)	.23	5.6 (4.0 to 7.1)	0 (–1.9 to 2.0)	.97	3.6 (2.4 to 4.8)	–0.4 (–1.7 to 2.4)	.74
2017	3.8 (2.6 to 5.0)			5.6 (3.7 to 7.5)			4.0 (2.3 to 5.6)		
≥2 chronic conditions									
2016	9.9 (9.3 to 10.6)	–1.4 (–2.1 to –0.8)	<.001	7.9 (7.2 to 8.6)	–0.4 (–1.1 to 0.2)	.15	7.7 (7.1 to 8.4)	–0.1 (–0.8 to 0.5)	.63
2017	8.5 (7.8 to 9.1)			7.5 (6.9 to 8.1)			7.6 (7.0 to 8.2)		

^a^ Outcomes measured during the last 30 days.

^b^ Paired-sample *t* test results.

**Figure 2.**
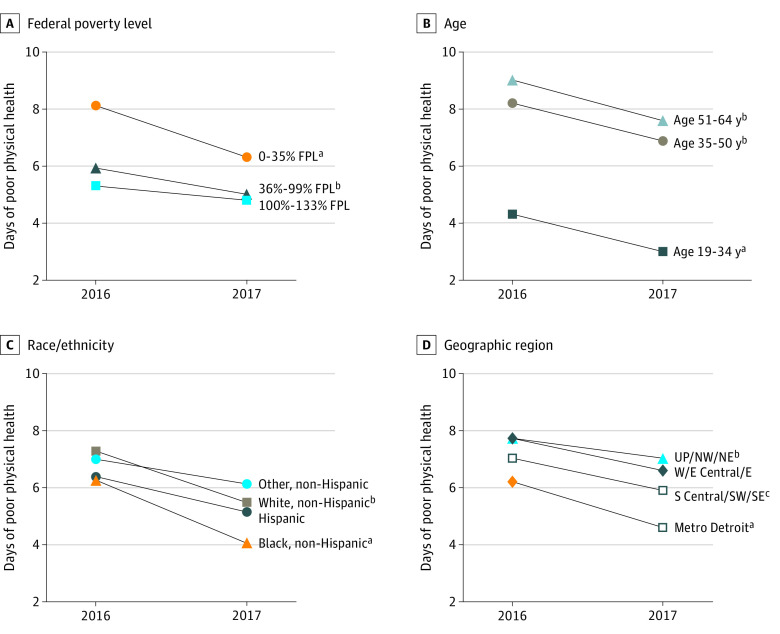
Changes in Days of Poor Physical Health From 2016 to 2017 Among All Enrollees by Select Demographic Characteristics E indicates Eastern Michigan; E Central, Eastern Central Michigan; FPL, federal poverty level; Metro, metropolitan; NE, Northeastern Michigan; NW, Northwestern Michigan; S Central, Southern Central Michigan; SE, Southeastern Michigan; SW, Southwestern Michigan; UP, Upper Peninsula; and W, Western Michigan. Paired-sample *t* test restricted to each group was used to test for a significant change over time. ^a^*P* < .001. ^b^*P* < .01. ^c^*P* < .05.

Significant reductions in reports of days of poor physical health from 2016 to 2017 were also observed among current and former enrollees who were non-Hispanic white (from 7.5 [95% CI, 7.0-8.1] to 6.1 [95% CI, 5.6-6.6]; *P* < .001), non-Hispanic black (from 5.9 [95% CI, 5.1-6.8] to 4.4 [95% CI, 3.6-5.1]; *P* < .001), from the Detroit metropolitan area (from 6.2 [95% CI, 5.4-6.9] to 4.6 [95% CI, 3.9-5.2]; *P* < .001), from Western and Eastern Central Michigan (from 7.7 [95% CI, 6.9-8.5] to 6.6 [95% CI, 5.9-7.3]; *P* = .004), from South Central, Southwestern, and Southeastern Michigan (from 7.0 [95% CI, 6.1-8.0] to 5.9 [95% CI, 5.1-6.8]; *P* = .02), and with an income of 0% to 35% of the FPL (from 8.1 [95% CI, 7.4-8.9] to 6.3 [95% CI, 5.7-7.0]; *P* < .001) and 36% to 99% of the FPL (from 5.9 [95% CI, 5.3-6.6] to 5.0 [95% CI, 4.4-5.6]; *P* = .009) (Figure 2). Significant reductions in reports of days of poor physical health were also found across all age groups (19-34 years, 4.3 [95% CI, 3.7-4.9] to 3.0 [95% CI, 2.5-3.5]; *P* < .001; 35-50 years, 8.2 [95% CI, 7.3-9.0] to 6.9 [95% CI, 6.1-7.7]; *P* = .002; 51-64 years, 9.0 [95% CI, 8.2-9.8] to 7.6 [95% CI, 6.9-8.3]; *P* = .001) (Figure 2).

## Discussion

This study is among the first, to our knowledge, to use a longitudinal panel survey of Medicaid expansion enrollees to assess the longer-term association with change in health after Medicaid expansion among historically marginalized groups. In this study, we found improved health for Medicaid expansion enrollees in Michigan from 2016 to 2017, including across all age groups and in populations with a long history of health disparities, such as non-Hispanic black individuals, those with incomes of less than 35% of the FPL, and those residing in the Detroit metropolitan area. Our findings align with those of other work using national data and different study designs (eg, secondary analysis of national data between expansion and nonexpansion states) that have shown improvements in health among racial/ethnic groups and chronic disease and behavioral health subgroups who gained Medicaid coverage.^10,16,29^ Our study is unique given the longitudinal panel nature of the design that allowed for assessment of the health status of actual Medicaid expansion enrollees to learn whether those who enrolled experienced improved health over time.

The Detroit metropolitan area has the largest Hispanic and non-Hispanic black population in the state of Michigan and saw its uninsured rate reduced by more than half as a result of the ACA insurance expansions.^30^ Improved health status among Detroit-area enrollees may be attributed to the ability to now access health care, coupled with more investments to the health care infrastructure in Detroit as a result of increased reimbursement from a more widely insured population.

Results document improvements in self-reported health status and days of poor physical health across all respondents and in critical subgroups such as those with chronic conditions, those with lower income, and non-Hispanic black respondents. Our findings support previous studies that have found Medicaid expansion to be associated with reductions in poor health days among chronically ill adults.^10,15^ Our findings also align with the randomized Oregon Health Insurance study that showed improvements in self-reported health in enrollees in the first 2 years after gaining Medicaid coverage.^31^ Policy interventions, such as expanding health insurance, are more likely to show health benefits and show them earlier when targeting those with poor health or a history of being uninsured or those with preexisting conditions.^15^

Therefore, what, specifically, might have improved health in Michigan’s Medicaid expansion? Its emphasis on primary care and the promotion of identification of health risks and health behaviors could have played a role. The HMP includes coverage for dental and vision services, both of which respond to enormous unmet needs in low-income populations and can have an important effect on perceived health. Enrollees may also benefit from other state initiatives aimed at improving the coordination and delivery of care that is supported through the Centers for Medicare & Medicaid Services State Innovation Model.^32^

In contrast to our physical health findings, we did not see significant changes in days of poor mental health or days of usual activities missed. Our findings differ from those of national serial cross-sectional studies, which found improvements in poor mental health days and activity limitations in Medicaid expansion states compared with nonexpansion states.^2^ The Oregon Health Insurance Study also found reduced depression in the first 2 years after gaining Medicaid coverage.^30^ Other research has shown that despite increases in coverage of services for mental health and substance abuse disorders,^33^ low rates of treatment remain a concern.^34^ Low-income individuals with behavioral health conditions may require high degrees of outreach and engagement to observe consistent improvements in mental health measures. These measures of mental health status may require longer longitudinal assessment or a focus on particular subgroups to observe improvements in self-reported mental health.

### Limitations

There are limitations to this study. Measurements of health status relied on individuals’ perceptions of their own health. Although reports of improved health from surveys may reflect social desirability or recall bias, self-report is considered a valid and reliable measure for assessing health, and our reports of change over time relied on assessing health status separately for each survey, rather than asking respondents to reflect on changes in health.^35^ The potential for bias due to sampling only those who consented to follow-up and also due to loss to follow-up, despite our strong consent and follow-up rates, is another limitation. Our analysis of nonresponse bias indicated little difference in demographic characteristics between those who did and did not complete the follow-up survey and weights adjusted for sampling and nonresponse bias. The subgroup that was no longer enrolled and uninsured was a small category, which may explain the wide CIs in those estimates. We measured changes between 2 short-term points. Longer-term follow-up of Medicaid expansion enrollees will be useful to confirm and extend our findings from this study. Our findings showed improvements in self-reported health among enrollees over time. Causal inference could not be determined from this observational study. Finally, state-specific contextual factors may limit generalizability of our findings to other states.

## Conclusions

This survey study found that enrollees in Michigan’s expanded Medicaid program, including racial/ethnic minorities, economically disadvantaged enrollees, those with chronic conditions, and those living in the Detroit metropolitan area, experienced improvements in self-reported health status and fewer days of poor physical health over time. Medicaid expansion enrollees are experiencing ongoing improvement in self-reported health status; these findings suggest that expansion supports national priorities of reducing health disparities for historically marginalized groups, a goal aligned with national and international health priorities.^19^

## Notes

Supplement. **eTable.** Comparison of Characteristics of 2017 Enrollee Follow-up Survey Respondents and Nonrespondents
