# Supplementary material for: Examination of Changes in Health Status Among Michigan Medicaid Expansion Enrollees From 2016 to 2017
Source: JAMA Netw Open. 2020 Jul 10;3(7):e208776. doi: 10.1001/jamanetworkopen.2020.8776 (PMC7352154; doi:10.1001/jamanetworkopen.2020.8776)
Supplement: Supplement. — eTable. Comparison of Characteristics of 2017 Enrollee Follow-up Survey Respondents and Nonrespondents [file jamanetwopen-3-e208776-s001.pdf]

Supplementary Online Content

Patel MR, Tipireni R, Kieffer EC, et al. Examination of changes in health status among Michigan Medicaid expansion enrollees from 2016 to 2017. *JAMA Netw Open*. 2020;3(7):e208776. doi:10.1001/jamanetworkopen.2020.8776

eTable. Comparison of Characteristics of 2017 Enrollee Follow-up Survey Respondents and Nonrespondents

This supplementary material has been provided by the authors to give readers additional information about their work.

©2020 Patel MR et al. *JAMA Network Open*.

eTable. Comparison of Characteristics of 2017 Enrollee Follow-up Survey Respondents and Nonrespondents

**Characteristics**	**Respondents (n=3,104) %**	**Nonrespondents (n=608) %**	***p* value**
**Age**			
19-34	40.1	49.7	<0.001
35-50	29.5	30.0	
51-64	30.5	20.3	
**Gender**			
Male	47.2	48.7	0.612
Female	52.8	51.3	
**Race/Ethnicity**			
Non-Hispanic White	60.3	60.8	0.881
Other	39.7	39.2	
**FPL category**			
0-35% FPL	53.1	45.6	0.018
36-99% FPL	27.7	32.1	
100-133% FPL	19.1	22.3	
**Interview language**			
Arabic	0.8	5.3	<0.001
English	98.7	93.6	
Spanish	0.5	1.2	
**Geographic Region**			
Northern Michigan	8.9	9.4	0.249
Central Michigan	29.3	24.8	
Southern Michigan	18.1	20.5	
Detroit Metro	43.7	45.3	

Notes: Chi-square test of independence

©2020 Patel MR et al. *JAMA Network Open*.
